# A Guide to Biodetection in Droplets

**DOI:** 10.1021/acs.analchem.3c04282

**Published:** 2024-06-06

**Authors:** Simona Bartkova, Marta Zapotoczna, Immanuel Sanka, Ott Scheler

**Affiliations:** † Department of Chemistry and Biotechnology, Tallinn University of Technology (TalTech), Akadeemia tee 15, Tallinn 12618, Estonia; ‡ Faculty of Biology, Biological and Chemical Research Centre, University of Warsaw, Żwirki i Wigury 101, 02-089 Warsaw, Poland

## Introduction

Encapsulation of biological samples into small (pico- and nanoliter) droplets allows physical and chemical separation and confinement, enabling high-throughput experiments at microscopic scale (e.g., single cell). The general workflow of droplet-based methods starts with encapsulation of a biosample in an aqueous solution, which is done under passive flow or in an active manner.^1^ Passive droplet generation relies only on the properties of the liquids or the geometry of the droplet generation device, e.g., T-junction in microfluidic chips.^1,2^ To ensure encapsulation of single cells, the input suspension is diluted to ca. 10-fold below the density of a cell per droplet volume to yield an emulsion of ca. 90% of empty droplets.^3^ On the other hand, active manipulation of liquids applies external actuation (e.g., magnetic field) ensuring the majority of droplets contain a biosample and that droplets can be split or merged with other droplets and reagents.^1,4^ The experimental workflow requires a target-specific label that interacts with the target, leading to a signal that can be detected and processed.

Droplet-based experiments offer immense resolution, high-throughput, and accelerated sample analyses.^5−7^ However, most experiments are still at the development stage with limited documentation and restricted know-how. Recent reviews discuss droplet-based applications in specific research fields^8−10^ or technical aspects and general advantages of droplet emulsion generation.^5,7,11^ In this tutorial, we provide guidance in selecting suitable detection labels (e.g., fluorescently tagged antibodies) for optical biodetection, as this is the most common approach for detecting and analyzing biological targets inside microdroplets. We focus on the following workflow aspects that are key for label selection, including biosample type, optical detection technique, detection target, label, and the signal (see description of aspects and complete workflow for each reviewed study in Table S1). Our guidance is based on recent studies that utilize state-of-the-art approaches for optical detection of biological samples in droplets, grouped in sections based on their study area.

## Nucleic Acid

**Figure 1:**
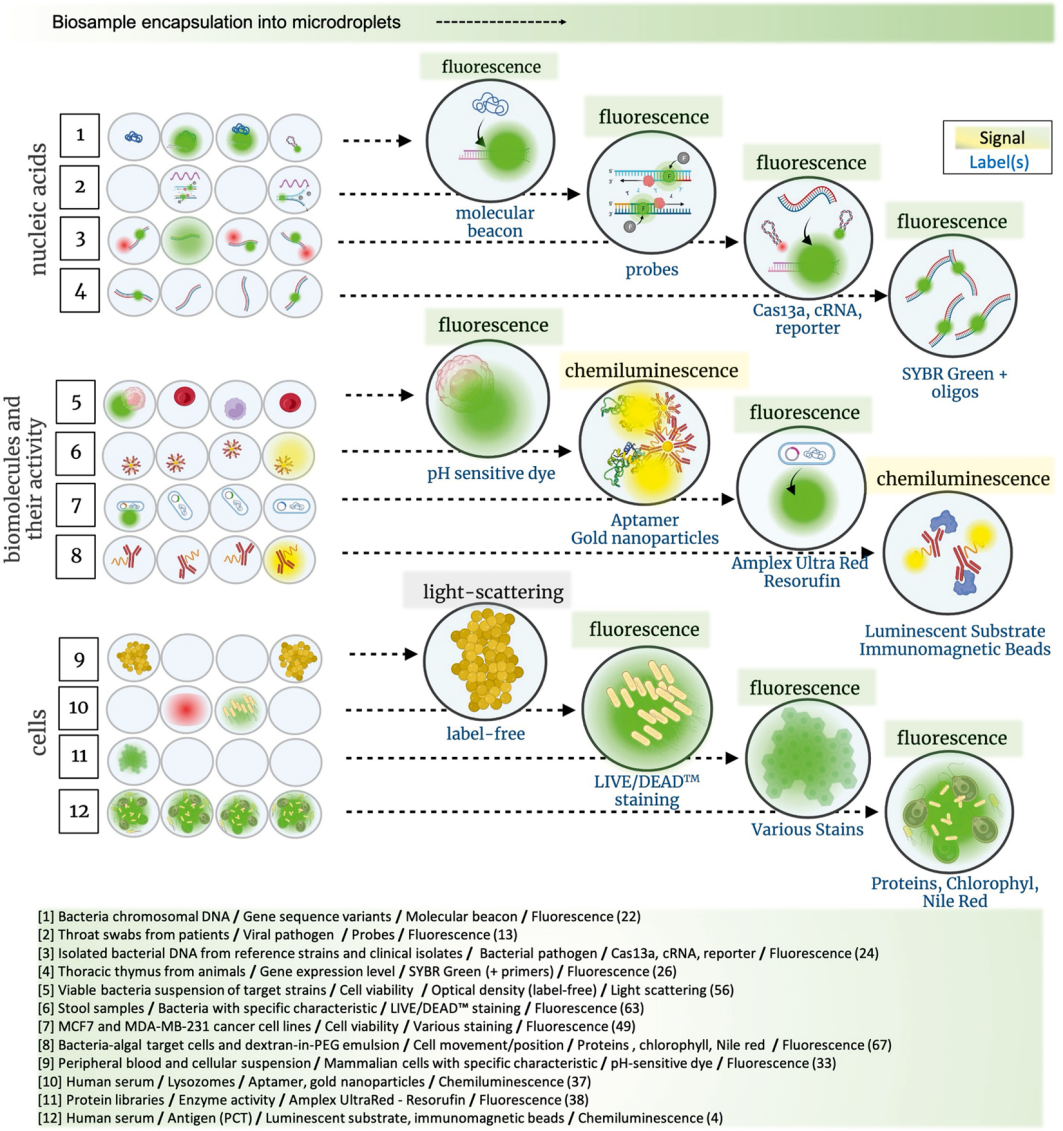
State-of-the-art signals and labels used in optical detection techniques for biological targets in microdroplet emulsions. All techniques are grouped together based on their main study area: (i) nucleic acid, (ii) cells, and (iii) biomolecules and their activity. Depicted examples are described by biosample/target/label/signal (reference).

**Figure 2:**
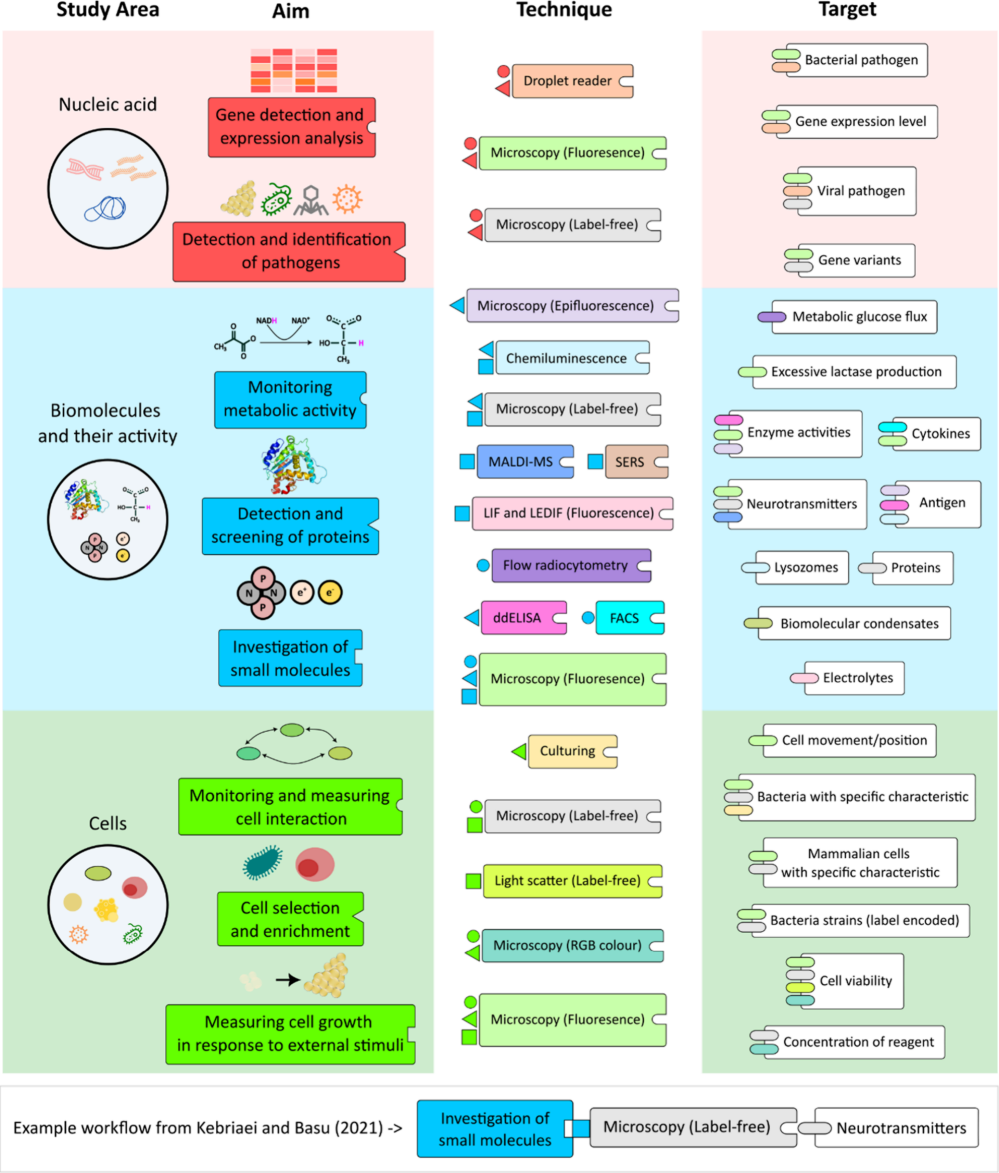
State-of-the-art workflow options for studying cells, molecules, and nucleic acids. The workflow options for the various study aims (e.g., measuring growth from single cells, metabolic activity, identification of pathogens) guide the choice of existing optical detection techniques as well as experimentally validated study targets. The workflow paths are connected in a lock and key fashion where the color of each step (box) and shape of their lock matches the color and shape of the key connected to the next possible step (box). Example workflow is shown from the study by Kebriaei and Basu.^41^

Detection of nucleic acids in droplets is performed via two approaches: (i) digital droplet polymerase chain reaction (ddPCR) and (ii) loop-mediated isothermal amplification method (LAMP), which produce an optical detection signal type. Both are primarily used for (i) detection and identification of pathogens or (ii) gene detection and expression analysis (see Figures 1 and 2). In ddPCR the reaction mixtures are partitioned into thousands of droplets which can be analyzed by Poisson distribution that approximates the probability distribution of positive reactions in each sample.^3^ Positive droplets produce a signal (e.g., fluorescence intensity) which is quantified via droplet reader devices as the optical detection technique (Table S1). In contrast, application of LAMP requires more advanced labels, but the workflow, including emulsification, is simplified (achieved via vortexing). For detection and quantification of signal intensity in droplets, fluorescence or label-free microscopy is used as the optical detection techniques (Table S1).^12^

### Detection and Identification of Pathogens

One robust tool for typing pathogens is ddPCR, as it has extremely low detection limits (5 × 10^4^ copy/reaction in comparison to 10^3^–10^7^ copies per reaction in RT-PCR) while minimizing cross-contamination.^13,14^ The ddPCR method has been used for the detection of RNA viruses, *e.g*., SARS-CoV-2 (Figure 1)^13^ and cucumber green mottle mosaic virus (CGMMV).^14^ The results of ddPCR amplification can be read by droplet readers, which require specific hardware. Fluorescence emission is enabled via fluorescent labels (e.g., FAM, TAMRA, HEX, and MGB). Due to robustness, methods based on ddPCR are rapidly disseminating despite the need to use specialized equipment, such as the droplet reader and accompanying droplet generators. However, one must be aware that most commercial ddPCR platforms are associated with initial high costs. Instead, Chi et al. coupled a novel method termed droplet DNAzyme-coupled rolling circle amplification (dDRC) with a fluorescence Naica Prism3 reader (droplet reader) to detect *Escherichia coli* in clinical urine samples with single-cell sensitivity.^15^ The method combines single-stranded DNA with enzymatic activity^16^ and a circular DNA template to produce long DNA or RNA sequences using short primer(s) at constant temperature.^17^ For the reaction, Chi et al. used a fluorogenic DNA-enzyme RFD-EC1 that cleaves target *E. coli* RNA into fragments containing Guanine-rich DNA sequences called repetitive G-quadruplexes.^15^ Fluorogenic dye Thioflavin T (ThT) binds to these sequences and produces a fluorescence signal. Chi et al. reported detection time for *E. coli* at ca. 90 min.^15^

LAMP is used for detection of viral and bacterial pathogens via fluorescence or label-free microscopy.^18,19^ In a proof-of-concept study, Fang et al. tested LAMP on samples containing Hepatitis B virus (HBV) nucleic acids.^18^ The workflow was based on an established droplet generation method using cross-linked PEG hydrogel followed by detection with fluorescence microscopy.^20^ The droplet hydrogel matrix enabled the amplification products to maintain spatial clustering, thus forming so-called polonies of identical amplicons. The polonies were detected as fluorescent signal (SYTOX Orange) intensity, corresponding with the heterogeneous amplification performance of individual molecules.^18^ Added restriction enzymes grouped identified genotypes by cleaving the nucleic acid at specific restriction sites, thereby preventing the double-looped DNA from further amplification (i.e., either small or no polony and low or no fluorescence signal). Fang et al. used fluorescence microscopy to further visualize the positive (genotype-specific) polonies with at least 3 × 10^5^ copies per reaction.^18^ In a different proof-of-concept study, LAMP was instead combined with label-free microscopy. Here, the main goal was to develop a deep learning algorithm for polydisperse droplet segmentation and target detection via bright-field microscopy. This would allow fast and simple, label-free detection of nucleic acids that would require only vortexing of the sample for polydisperse droplet generation following a bright-field microscope for imaging. Chen et al. aimed to improve LAMP detection via bright field microscopy by implementing a deep-learning algorithm for the image analysis.^19^ Optical detection via bright-field was compared to fluorescence microscopy, while viral pathogen SARS-CoV-2 and bacterial pathogen *Proteus mirabilis* were used as detection targets. Fluorescence dye SYBR green and precipitated magnesium pyrophosphate byproducts (occurs naturally in LAMP reactions) were used as labels for the target nucleic acid. Fluorescence dyes FITC and TRITC conjugated to IgG antibodies were used to further examine polydisperse droplet coalescence (verify whether droplets remained stable). Using a deep-learning algorithm for analysis, they acquired a lower limit of detection (down to 5.6 copies per μL of target nucleic acid) via bright-field microscopy than with fluorescence microscopy.^19^ In a study performed by Azizi et al., LAMP was used for detection of *Salmonella typhimurium*in contaminated milk.^21^ Detection of RNA increased the method sensitivity due to a higher copy number in bacterial lysate at 5 × 10^5^ CFU per mL of bacteria, a number 2-fold lower than the detection limit of pure bacterial culture. Increased droplet volume and incubation time resulted in lower detection limit.^21^ Moreover, incorporation of the sequence specific molecular beacons (MBs) into the platform called MB-ddLAMP-PCR enabled detection of single-nucleotide polymorphisms, thus distinguishing the *ompW* gene that encodes the outer membrane protein variants from bacteria *Vibrio cholera*(22) (Figure 1). Authors proposed usage of a customized portable 3D printed droplet generator with modified fluorescence and label-free microscopy techniques. Analysis could then be facilitated by a smartphone camera and microscope. The detection limit was down to 4.39 copies per μL of reaction.^22^

Specific detection of nucleic acids has been achieved using other custom platforms.^23,24^ One such platform called TriD-LAMP (duplex droplet dLAMP) is highlighted in the works of Wu et al. Here, droplets are generated through Laplace pressure using 64 parallel nozzles, which can generate thousands of droplets with low CV%.^23^ Wu et al. used the platform combined with fluorescence microscopy to simultaneously detect two targets: the bacterial pathogen *E. coli* and the bacteriophage λ.^23^ This is possible with two different fluorescence labels, e.g., FAM and HEX. The platform is compatible with recombinase polymerase amplification (RPA), nucleic acid sequence-based amplification (NASBA), and rolling circle amplification (RCA). Another multiplex platform developed by Thakku et al. called DropArray platform (Figure 1)^24^ is an expansion of a previous droplet platform called CARMEN.^25^ The platform Bacterial Combinatorial Arrayed Reactions for Multiplexed Evaluation of Nucleic Acids (bCARMEN) uses fluorescence microscopy for classification of 52 different bacterial pathogens. To improve the sensitivity and specificity, the authors used combinatorial pairwise experiments of encapsulated preamplified nucleic acid targets and Cas13-guided detection. They found that the CRISPR RNAs enhanced the discriminatory power of this diagnostic platform. bCARMEN was shown to be suitable as a point-of-care diagnostic test.^24^

### Gene Detection and Expression Analysis

Sample partitioning into droplets containing different reactions enhances massive parallelization, which is important for samples limited in volume or containing low concentration of nucleic acids.^24,26−29^ Divari et al. 2022 (Figure 1) used ddPCR to study expression genes encoding hormones in livestock animals to monitor the illegal use of supplements. The *FKBP5* (FKBP prolyl isomerase 5) gene expression in bovine thymus is regulated by glucocorticoids and thus was used as the target.^26^ Commercially available dsDNA binding dye (EvaGreen) was used to label the target (specific amplicons) following fluorescence intensity readouts. Sensitivity of the assay was estimated at 0.05 ng per μL of cDNA for the *FKBP5* target gene and 1 copy per μL of a reference gene. To simplify multiprotein expression studies, Sierra et al. utilized Streptavidin-coated polystyrene beads for binding/coating target DNA.^27^ This enabled the creation of cell-free expression systems inside droplets. The high-throughput method manifested by protein synthesis is facilitated by fluorescence detection and builds upon previously used standard methods reported by Plesa et al.^30^ and Sidore et al.^31^ In this study, rapid testing of multigene programs was enabled via droplets using Golden Gate (BsaI and T4 ligase) and single-enzyme DNA recombination (BxB1 integrase). The gene expression level was detected and quantified via a fluorescence emission signal from biotinylated DNA encoding mScarlet, mNeonGreen, and LSSmOrange fluorescence labels. Overall, this cell-free system eliminates cloning and could potentially accelerate the design–build–test cycle in synthetic biology as reported by Sierra et al.^27^ Droplet encapsulation coupled with fluorescence detection can also enhance detection and expression level measurement of specific target mRNA transcripts from single cells, as exemplified by the recent works of Hyman et al.^28^ The invented droplet platform is called single-cell nucleic acid profiling in droplets (SNAPD) and was used for analyzing transcriptional biomarkers in thousands of single mammalian cells. By incorporating LAMP amplification and multiple universal labels such as FAM, HEX, and SYBR green, Hyman et al. could perform a multiplex assay where fluorescence signals were based on specific target gene sequences of the biomarkers.^28^ The authors tested this approach by detecting 60S Ribosomal Protein L3 (RPL3) in the leukocytic leukemia line (MOLT-4), HER2 (*ERBB2*) of breast cancer marker, mesenchymal marker vimentin (*VIM*), and epithelial marker cytokeratin 19 (*KRT19*). Another study by Clark et al. also utilized droplet-based microfluidics with fluorescence microscopy detection for targeting specific mRNA transcripts.^29^ Two genes, *Aqp4* and *Edem1*, were chosen as target biomarkers for detecting transcription factor XBP1 activation in astrocytes, which promote disease pathology in multiple sclerosis and experimental autoimmune encephalomyelitis. The state-of-the-art platform single-cell FIND-seq (scFIND-seq) developed by Clark et al. enabled analysis of millions of cells in a cost-effective manner compared with scRNA-seq.^29^ The authors were able to trap both genome and transcriptome together in molten agarose droplets enabling reverse transcription of cDNA. Solidified agarose beads were then reinjected into the microfluidic device for digital PCR amplification where SYBR green and multiplexed TaqMan probes were used for labeling cells expressing the target RNA transcript. The fluorescence signal enabled sorting via a previously developed dielectrophoretic microfluidic device,^32^ and target transcripts could then be sequenced. Another novel droplet platform called bCARMEN (described in detail in the previous section) used by Thakku et al. for bacterial pathogen detection via fluorescence technique (Figure 1) was further utilized to detect specific antimicrobial resistant (AMR) genes.^24^ Exactly the same approach was taken by using the RNase Alert v2 kit along with crRNAs complementary to the AMR target gene sequences.^24^

## Biomolecules and Their Activity

Approaches developed for detection of biomolecules and their activity, including proteins and small molecules, utilize several different optical detection techniques (Table S1) that mainly focus on fluorescence and chemiluminescence detection signals (Figure 2). The choice of specific labels for studied targets is limited and often requires custom development. A variety of relevant experimental procedures have been developed in recent years offering increased speed, lower cost, and higher resolution and sensitivity.

### Monitoring Metabolic Activity

Metabolic activity is a good indicator of cellular disorders. For instance, elevated glycolysis resulting in excessive lactate production is a long-known property of metabolically active Circulating Tumor Cells (CTCs). Radfar et al. demonstrated the ability to effectively detect CTCs by utilizing the rapid acidification inside droplets caused by excessive lactate production of the CTCs.^33^ This was done using a pH-sensitive fluorescent dye as a label in a point-of-care device. Operation of the device could be done with a simple pipet with novel arrow-shape channels enabling encapsulation of single cells (Figure 1).^33^ This nonspecialized setup made the method relevant for clinical applicability by reducing the time of sample processing and cost, making it an attractive platform for studies of metabolic activity that is associated with a pH reduction.

The measurement of glucose flux in single cells has been achieved by flow radiocytometry (FMCR).^34^ The FMCR measures the heterogeneous behavior of single cells on the basis of the transport and incorporation of a radiolabeled substrate, such as the fluorine-18-labeled glucose analogue. The substrate is a well-known radiotracer in positron emission tomography, routinely used to detect metabolically active malignant lesions in diagnosis of a range of cancer types.^35^ The method consists of high-throughput radiometry of single cells, followed by downstream cell sorting. FMCR is similar to flow cytometry, but involves molecular probes that convert to fluorophores in response to ionizing radiation (radioactive signals).^34^ First, radiolabeled single cells were encapsulated with radiofluorogenic probes in water-in-oil drops emitting ionizing particles and producing reactive oxygen species (ROS) via water radiolysis. These ROS instantaneously reacted with radiofluorogenic probes, generating a fluorescent signal proportional to the level of radioactivity. By converting the stochastic radioactive decays into a continuous fluorescence signal, the FMCR significantly reduced (300-fold) the exposure time per cell, reaching 500 single cells below a minute. To validate the method, the metabolic flux of glucose was effectively measured in thousands of single breast cancer cells after incubation with the radiotracer.^34^

### Detection and Screening of Proteins

Droplet encapsulation together with fluorescence techniques have been used to improve detection of biomolecules such as proteins secreted by single cells.^36^ Yuan et al. used a microfluidic workflow for coencapsulation of single natural killer NK-92 MI cells and their target K562 cells, consisting of (i) in-droplet cytokine IFN-γ capture assays with specific fluorescent anti-IFN-γ antibody for labeling and (ii) detection of the activated IFN-γ producing NK-92 MI cells via fluorescence microscopy and flow cytometry (FACS).^36^ The activated cells represent a fraction of the large population; thus, methods in the bulk may be insufficient for detection. By intsead compartmentalizing cells into droplets, diffusion of secreted cytokines to neighboring cells is prevented and reduces false positives and false negatives.^36^ FACs sorting of the droplet released activated NK cells can be expanded in culture for further functional studies and characterization.^36^

Giuffrida et al. reported a method of lysozyme biosensing with gold nanoparticles (AuNPs).^37^ Integration of the AuNPs-enhanced CL detection in droplet microfluidic devices, combined with the aptamer-driven specific adsorption of lysozome on the AuNPs surface, was used to detect lysozome concentration as low as 44.6 fM, in sample volumes as low as 1 μL, and in a rapid assay time of only 20 min (Figure 1). The particles of antilysozyme aptamers (apt-AuNPs) were evaluated using blood and serum samples, validating their high specificity.^37^ Next, an assay that enables fluorescence detection of enzymatic activity through a reaction cascade for dehydrogenases was developed to screen for enzymatic variants with improved activity (Figure 1). Kolaitis et al. proposed applying a hydrogen peroxide-forming NADH oxidase coupled with peroxidase-catalyzed fluorescence generation to quantify NADH levels corresponding to dehydrogenase activity (Figure 1).^38^ They explored the utility of this assay in the evolution of an alcohol dehydrogenase from *Sphingomonas* species A1 (SpsADH). A fluorescence-activated droplet sorting platform was used to screen 50,000 variants of SpsADH libraries toward the non-native substrate L-guluronate with the potential to serve as raw material for the biobased production of chemicals. A variant with a 2.6-fold improvement in catalytic efficiency *k*_cat_/*K*_m_ toward the non-native substrate was identified.^38^ A point-of-care, wash-free, and single-step digital droplet immunoassay was developed using biomarker interleukin-8 (IL-8) as an example analyte.^39^ To reduce the number of steps, a so-called proximity ligation chemistry was used. Proximity-based assays use two or more reaction components coming into proximity for a signal to be generated; here, they are two antibodies specific to proximal parts of the antigen. Upon binding of both (the target antigen is recognized by the antibody), the reaction components are in close enough proximity to cause a secondary reaction (ligation of oligonucleotide fragments associated with the antibodies) that results in a signal (jointed DNA fragments become a template for gene expression), here fluorescence. The simplicity of this “one-pot” method is further reflected in the emulsification (production of aqueous droplets) by shaking and mixing the aqueous and oil phases to produce polydisperse droplets containing the complete reaction mix. The sensitivity of the method was reported as low-pM limits of detection.^39^ Droplet digital ELISA (ddELISA) developed by Cohen et al. enabled detection of a potential cancer biomarker LINE1/ORF1, which had never been measured in serum.^40^ The principle of ddELISA is based on the Simoa method, where antibody coated paramagnetic beads are added to the sample containing the target antigen. A key factor is addition of fluorescence label fluorescein di-β-D-galactopyranoside (FDG) to enable optimal detection of droplets containing the target. This enabled an approximate 25-fold increase in sensitivity over that of the gold standard Simoa.

Label-free techniques can provide universal detection of proteins by exploiting the fundamental physicochemical property of proteins which adsorb to a liquid–liquid interface due to their hydrophilic and hydrophobic regions.^41^ The adsorption impacts the dynamic interfacial tension of an immiscible (water–oil) interface, which is used in separation of proteins such as in axisymmetric drop shape analysis. Fluctuations in protein concentration alter the size and shape of the droplets, and these changes can be quantified using an inverted microscope, a high-speed camera, and in-house imaging software. Two applications of the method were demonstrated: (1) direct injection of a single protein into a microfluidic chip and (2) postcolumn detection of protein mixtures separated by high-performance size exclusion chromatography. The lowest detection limit without an HPLC of approximately 1 μg/mL thyroglobulin protein in 1 nL droplet corresponded to 1 fg total protein. The aforementioned detection method used as a detector offered a sensitivity 6 orders of magnitude higher than conventional UV–vis detectors.^41^

### Investigation of Small Molecules

Detection of metabolites is complicated, usually requiring coupling of droplet microfluidics with other state-of-the-art technologies such as laser-induced fluorescence,^42^ single-droplet surface-enhanced Raman scattering (SERS),^43^ and chemiluminescence immunoassay (CLIA)^4^ (Figure 2). Wang et al. reported a method for selective and sensitive fluorescence-based ion-sensing methodology via droplet microfluidics.^42^ The oil stream was mixed with sensor ingredients, including an ionophore, a cation exchanger, and a permanently cationic fluorophore as the label. Electrolytes from the aqueous sample were extracted into oil segments, and the cationic dyes were displaced into aqueous droplets. Laser-induced fluorescence of the two immiscible phases was collected alternately without mixing the phases. The cation exchanger tetrakis[3,5-bis(trifluoromethyl)phenyl]borate enhanced the dye emission in the nonpolar sensing oil by preventing ion-pairing interactions and aggregations of the dye molecules, thereby increasing the detection limit to low concentrations of sensing chemicals (10 μM) in the oil. Using valinomycin as the ionophore and methylene blue as the dye, K^+^ is detected with a response time of approximately 11 s, 20-fold total fluorescence response, more than 1000-fold selectivity against other electrolyte cations, and without cross-sensitivity toward the sample pH. The method allowed for successful determination of K^+^ concentration in undiluted whole blood and sweat samples. Detection of other ionic analytes such as Ca^2+^ can be achieved using the corresponding ionophores.^42^ Biomolecular condensates are also molecules that are currently receiving much attention due to their critical functions within eukaryotic cells and their possible transition into aggregates linked to neurodegenerative diseases. In order to study the molecular details of such condensates, Avni et al., developed an ultrasensitive SERS method.^43^ Via inclusion of droplet technology, samples could be encapsulated with surface functionalized silver nanoparticles in liquid droplets, which enhanced the Raman signal.^43^ This enabled investigation of Fused in Sarcoma (FUS) condensates, which are some of the best prototypes of phase-separating proteins, with never before seen sensitivity. For labeling of FUS, Avni et al. used fluorescence dyes AlexaFluor488-C5-maleimide and Fluorescein-5-maleimide.^43^ Silver nanoparticles were iodide-modified (Ag IMNPs), allowing electrostatic interaction between the negatively charged Ag IMNPs and positively charged polypeptide chains of FUS inside each droplet. This caused significant plasmonic enhancement of specific protein vibrational modes, i.e., increased sensitivity, and enabled in-depth study of the molecular heterogeneity in FUS condensates.^43^ Nevertheless, the hardware needed for this method is far from available in every lab. Other clinically important molecules include biomarkers whose detection via immunochemical reactions enables identification of infections and implementation of correct treatment. One such biomarker is the peptide precursor procalcitonin (PCT) that can help identify sepsis and serious bacterial infections. Huang et al. combined chemiluminescence immunoassay (CLIA)^44^ with an active droplet-array (ADA) microfluidic approach^45^ for developing a novel microfluidics-based CLIA system, consisting of a compact microchip analyzer and microfluidic chips with preloaded reagents.^4^ The entire workflow of this CLIA system is automated, enabling detection time of PCT in only 12 min. The CLIA consists of four main steps all enabled by droplets being moved around via magnetic actuation: (i) Encapsulation of the antigen target protein (PCT) and antibodies conjugated to carboxylated immunomagnetic beads (CIMBs) into droplets where the antigen binds to the antibodies; (ii) droplets are moved into another chamber and merged with enzyme-labeled antibody (detection antibody) that enables formation of a double antibody sandwich; (iii) CIMBs rinsing; and (iv) droplet transfer into the final chamber and merging with chemiluminescent substrate APS-5 substrate enabling the enzymatic chemiluminescence (Figure 1).^4^

Neurotransmitters are also frequently studied molecules as they can potentially improve prevention of neurodegenerative diseases.^46,47^ One such neurotransmitter is γ-aminobutyric acid (GABA), whose levels are associated with many medical conditions. Bell et al. investigated whether usage of droplet microfluidics in association with offline matrix-assisted laser desorption/ionization-mass spectrometry (MALDI)-MS could be an optimal tool for label-free analysis of such small molecules as GABA.^46^ The workflow started with the generation of droplets with GABA that were deposited onto indium–tin oxide coated glass slides. Droplets evaporated, and microscopy imaging and Raman spectroscopy were used for studying droplet morphology and localization on the slide of the dried droplets, respectively. Finally, MALDI-MS imaging of the dried droplets was performed for detecting and quantifying concentrations of GABA. One crucial parameter producing the best GABA signal was the type of oil phase used for droplet generation, which Bell et al. deemed to be oil phases containing FC-40: perfluorooctanol (PFO) (10:1 v/v). This enabled having down to 23 amol limit of detection for GABA.^46^ This method does, however, require an extended amount of specific instrumentation, e.g., MALDI TOF/TOF mass spectrometer and specialized microscopy such as Raman. Another well-studied neurotransmitter is dopamine. The study by Alizadeh et al. demonstrated use of polymer dots (Pdots) as labels for fluorescence microscopy of dopamine in droplet-encapsulated single dopaminergic neuron PC12 cells.^47^ Pdots were synthesized using a solution of urea, neutral red, and trisodium citrate heated hydrothermally. In the method they functioned as the fluorometric reporter (label), while the easily oxidized dopamine^48^ served as the fluorescence quencher of the Pdot fluorescence due to the so-called inner filter effect. The fluorescence intensities decreased with increasing successive aliquots of dopamine concentrations from (1 nM to 900 μM).^47^ The detection of dopamine was moreover shown to be specific with no interference from other molecules such as ascorbic acid, uric acid, glutathione, glucose, epinephrine, etc.^47^

## Cells

Optical detection techniques used in studies of cellular phenotypes or interactions between various cell types include fluorescence and label-free microscopy (Figure 2 and Table S1). The choice of the most suitable label is dependent on the detection target and is related to the study aim, as discussed below.

### Measurement of Cellular Growth

The most popular labels for measurement of cellular growth in droplets are fluorescent viability markers followed by fluorescence measurement with a microscope.^49,50^ Byrnes et al. used a fluorogenic substrate (4-methylumbelliferyl-ß-d-glucuronide or MUG) for measuring glucuronidase activity; a known characteristic that happens during division of 94–96% *E. coli*.^50^ They were able to correlate the fluorescence intensity with *E. coli* growth. On the other hand, Seeto et al. used different labeling strategies, including (i) Live/Dead viability kit (Invitrogen) with calcein AM and ethidium homodimer 2 and (ii) CellTiter-Glo 3D luminescent cell viability assay (Promega) that is specifically used for 3D cultures and measures viability based on luminescence from ATP.^49^ For metabolic activity, 23-bis(2-methoxy-4-nitro-5-sulphenyl)-(2H)-tetrazolium-5-carboxanilide (XXT) was detected, as it turns into an orange formazan product via cell respiration (a redox potential reaction). For investigating morphology and proliferation of cells inside droplets, two different targets were utilized via immunostaining: (i) fluorescence labels Alexa Fluor 488 and Alexa Fluor 568 Phalloidin conjugated with specific antibodies and (ii) Hoechst 33342 dye that has low toxicity and good cell permeability for visualizing the nucleus of living cells. The monodisperse microspheres used for encapsulating cancer cells were made from biosynthetic hybrid hydrogels composed of poly(ethylene glycol diacrylate) (PEGDA) covalently conjugated to natural protein (fibrinogen) (PEG-fibrinogen, PF). This enabled Seeto et al. to not only perform high-throughput drug screening but also simultaneously study cancer cells in 3D culture and the cell tumorigenic characterization.^49^ One potential disadvantage of the use of fluorescence dyes is the risk of their leakage out of the droplets, especially after prolonged incubations.^51^

Mahler et al. used reporter (mCherry and mKATE) bacteria to screen for a novel antimicrobial compound in soil communities.^52^ The reporter strains were pico-injected into monodisperse droplets with preincubated soil microbes and sorted based on fluorescence intensities upon incubation.^52^ Label-free microscopy has been demonstrated as an alternative detection technique in antimicrobial resistance testing (AST) and minimal inhibitory concentration (MIC) at the single cell level.^53,54^ Substituting fluorescence with color coding (RGB) allowed Svensson et al. and Jeong et al. to perform multiplex screening of antibiotic concentrations without the issue of fluorescence overlap.^53,54^ Svensson et al. applied colored polystyrene beads to represent antibiotic concentrations (up to 36 codes) and coencapsulated them with *E. coli* into monodisperse droplets.^53^ Visualization included a low-cost stereomicroscope, and 8 h of incubation was needed to distinguish growing bacteria, which is faster than common plating methods. Similarly, Jeong et al. used food dye as representation for different antibiotics and concentrations and coencapsulated *E. coli* with three clinically important antibiotics and three concentrations for validation.^54^ For visualization, a CCD was enough to capture the RGB colors, yet incubation time was 16 h and additional image processing with filtering and calculations was also required.

Light scattering via optical fiber is a novel label-free approach for measuring bacterial viability in droplets.^55,56^ The optical intensity of the scattered light correlates to bacterial density in the sample, allowing quantification of bacteria cells. Minimum incubation time for determining single-cell inhibitory concentration (scMIC) was 5 h.^56^ The scattering platform was then applied with fluorescence enabling differentiation of the species and investigation of scMIC profiles of complex bacterial populations (Figure 1).^55^ Limitations include detection of bacteria with filamentous or aggregating phenotype or slow growth and the need for special advanced hardware. Moreover, some antibiotic compounds are prone to leak out of droplets.^57^ This has to be taken into account during initial experimental procedure setup and subsequent evaluation of data results.

### Cell Selection and Enrichment

The broad range of available fluorescent labels translates into the popularity of the use of fluorescence in cellular enrichment applications.^28,33^ An example is SNAPD^28^ described in more detail in the section Nucleic Acid. In short, LAMP amplification, fluorescence detection, and fluorescence labels (e.g., FAM, HEX, SYBR green) incorporated into the droplet platform enabled selection of specific clinically relevant genetic markers (target) of mammalian cells. Fluorescence is also used for enrichment and tracking of bacterial cells.^58−60^ Taylor et al. performed high-throughput monitoring of stochastic bacterial growth trajectories of small populations using an adapted particle tracking algorithm.^58^ These were *E. coli* producing yellow fluorescence protein, which enabled counting individual bacterial cells inside droplets over time. Villa et al. utilized green fluorescence protein (GFP) producing *E. coli* for initial validation of a newly developed platform called MicDrop, which would improve selecting bacterial species that can degrade complex dietary carbohydrates from human gut microbiota.^59^ Monitoring fluorescence emission of the strain ensured that the single cells of bacteria could indeed be separated into individual droplets and replicate within the droplets for at least 5 days. Subsequently, Villa et al. tested MicDrop on fresh stool samples.^59^ This included droplet encapsulation followed by droplet incubation in an anaerobic chamber and finally qPCR and 16S rRNA sequencing. The method revealed that all test subjects possessed gut bacterial species capable of degrading common dietary polysaccharides. In congruence with previous research,^61^ the method also highlighted that a greater taxonomic richness can be seen via isolation and culturing of human fecal microbiota in droplets versus growth via traditional bulk conditions. In support, another study utilizing encapsulation of gut microbiota into droplets also showed enrichment of the sample and improvement of species-specific selection.^60^ Here, two different labels were used: (i) fluorescent reference beads (polystyrene colloids) that were mixed with sample to measure cell density and (ii) frequently used fluorescence protein FAM combined with specific TaqMan probes that are cleaved and allow FAM to emit a fluorescence signal if target DNA is present. The platform developed by Pryzlak et al. enabled visualization via fluorescence microscopy as well as sorting via electrodes after PCR, thereby selecting only target species for further processing such as quality whole genome sequencing.^60^ The platform nevertheless requires sophisticated hardware to analyze droplet fluorescence.

Droplet generation is moreover often combined with culturing techniques, as initial encapsulation in droplets allows competitive-free single-cell growth and subsequent culturing on agar plates allows further selection and manipulation such as sequencing.^52,62,63^ Mahler et al. combined single-cell droplet encapsulation with culturing on agar plates for enrichment of complex soil communities.^52^ The droplets with cells were inserted into a capillary mounted on a positioning system that allowed continuous dripping of droplets onto a spiral-patterned moving agar plate. Mahler et al. further combined culturing and fluorescence microscopy for screening bacteria with possible antimicrobial properties within soil communities.^52^ Details regarding this are covered in a previous section. Screening for antimicrobial resistance can also be enhanced via droplet encapsulation and culturing.^62^ This enabled Watterson et al. to enrich and select for slow-growing antibiotic-resistant gut bacteria from human fecal samples.^62^ In another study, Yin et al. also encapsulated single cells from fecal samples to enrich slow-growing gut bacterial species; however, the goal in this study was to select for cells with antiobesity potential (Figure 1).^63^ To validate that the cell density in fecal samples would ensure single-cell droplet formation, Yin et al. initially tested their droplet microfluidic setup by encapsulating GFP transformed *E. coli* BL21 and monitoring droplets via fluorescence microscopy.^63^ For measuring viability of cells in fecal samples before droplet encapsulation, Yin et al. performed staining with the Live/Dead BacLight Bacterial Viability kit (Invitrogen).^63^ The kit contains SYTO 9 that labels nuclei of all cells and propidium iodide that only enters and stains dead cells as it is cell impermeant. After the incubation of droplets with sample cells, the droplets were cultured in an anaerobic chamber on agar plates with selective media metabolites (supernatant) from engineered butyrate-producing bacteria (EBPB). Only desired bacteria would form colonies, which could be sequenced thereafter.^63^

Label-free microscopy is another previously used technique for enrichment and selection of cells, yet not as often employed as fluorescence and culturing.^62,64^ In the study by Watterson et al., culturing was combined with an additional image-based sorting algorithm and microfluidic control system allowing the sorting of droplets based on the encapsulated bacterial colony density.^62^ Antibiotics were coencapsulated with bacteria, allowing only resistant bacteria to grow inside droplets. Subsequent sequencing of the sorted droplets then revealed genomic details of the bacterial species and their resistance profiles. Zielke et al. applied label-free microscopy with passive sorting to isolate activated T-cells.^64^ The custom developed sorting platform named “Sorting by Interfacial Tension” (SIFT) utilized the fact that activated T-cells have increased glycolysis and thereby lower the pH in their droplets versus droplets containing naive cells. Change in droplet pH can lead to a concurrent increase in droplet interfacial tension. Sorting is thus achieved by droplets with activated T-cells and high glycolysis production being flattened and displaced when they encounter a microfabricated trench within the SIFT microfluidic platform.

### Monitoring and Measuring Cell Interaction

The dominant technique for all droplet-based studies involving cell interaction is fluorescence microscopy.^65,66^ To study bacterial networks under various conditions, Hsu et al. developed a droplet platform termed Microbial Interaction Network Inference in microdroplets (MINI-Drop).^65^ For evaluating the accuracy and dynamic range of the cell counting method, Hsu et al. used several fluorescence protein labels incorporated into bacteria strains (i.e., CFP-labeled *E. coli*, RFP-labeled *E. coli*, and YFP-labeled *S. typhimurium*).^65^ To further investigate the interaction network with MINI-Drop, Hsu et al. constructed a synthetic consortium with fluorescence protein labels composed of RFP-labeled *E. coli* methionine auxotroph (EC Met-) and a GFP-labeled *B. subtilis* tryptophan auxotroph.^65^ The consortia were coencapsulated in droplets with various media to observe if it was possible to create a bidirectional positive interaction network. By coupling fluorescence microscopy to computer vision, each strain could be followed and counted in hundreds to thousands of droplets per condition. Tan et al. also used fluorescence microscopy to evaluate whether droplet size has an effect on syntrophic interactions of coencapsulated bacteria.^66^ Two *E. coli* with constitutively expressed fluorescent protein reporters, mNeonGreen and mCherry, respectively, were tested. Besides encapsulation in different sized droplets, interactions were also modulated through supplementation of amino acids in the medium.^66^ As hypothesized, the authors highlighted that the droplet size matters when studying interactions affecting the growth capacity, maximum specific growth rate, and lag time, depending on the degree of the interaction.

In the field of biotechnology and synthetic biology, fluorescence can be utilized for creating complex droplet microreactors where different species will spontaneously organize into specific structures enabling them to work in synergy.^67^ For enabling this, Xu et al. constructed complex polydisperse droplets consisting of (i) aqueous two-phase separated dextran-in-PEG and (ii) synthesized microparticles of denatured bovine serum albumin protein (BSA).^67^ Via shearing, they successfully created special microreactors capable of aerobic (oxygen producing) and hypoxic (hydrogen producing) photosynthesis in daylight and under aerobic conditions. Xu et al. coencapsulated algal cells or algal with nonphotosynthetic bacterial cells which were spontaneously organized and immobilized in different parts of these microreactors (Figure 1).^67^ To monitor whether species were correctly organized spatially in the droplets, the authors used fluorescence protein Atto425 for tagging *E. coli* and the naturally produced fluorescence of algal cells in the form of chlorophyll. To moreover visualize whether the microreactors were constructed correctly, Xu et al. used fluorescein isothiocyanate for the dextran portion of the droplets and specific target labeling for BSA particles in the form of the lipophilic stain Nile Red that fluoresces in lipid rich environments.^67^

## Conclusion

Using optical detection techniques has provided reliable workflow and different throughput^68^ options for studies of various biological samples. Studies mostly acquired experimental data by modifying or combining already existing standard and/or state-of-the-art approches^14,47,52^ or developing novel custom-made droplet platforms.^4,24,65^ This enabled studying a wide range of targets with high resolution, such as mammalian cells, bacteria, viruses, proteins, DNA, RNA, antibodies, metabolites, etc. (Figure 2). Most frequently used labels were universal (e.g., GFP, FYP, RFP, SYBR green, etc.) and specific (e.g., live/dead cell staining, coated particles, molecular probes, etc.) fluorescence-based labels (Table S1). Nevertheless, there are two main drawbacks accompanied by use of fluorescence labels for droplet emulsion experiments: (i) Fluorescence signals can overlap spectrally,^69^ causing issues if one has highly heterogeneous targets to detect or need to encode many different experimental conditions. (ii) Fluorescence dyes can leak out into the oil phase and neighboring droplets such as seen with the frequently used resorufin.^51^ To solve overlapping signals, one solution could be implementing unmixing algorithms during analysis such as PICASSO developed by Seo et al.^70^ To troubleshoot possible substrate or product leakage, Gantz et al. discussed methodological options in section nine of their review.^68^ Therein Gantz et al. also offered advice for reducing droplet evaporation, such as storage in a closed system.^68^ Another possibility for reducing leakage could be following a novel analytical approach proposed by Zinchenko et al.^71^ or alternatively use fluorescent nanoparticles instead. Carbon-based nanoparticles are especially gaining popularity as they are biocompatible, small in size, and show low toxicity.^47^ An overall ideal solution would be to omit fluorescence by use of label-free optical detection as exhibited by several studies covered in this review.^41,54−56^ Nevertheless, the main drawback with label-free detection in droplet emulsions that researchers should be aware of is the need to often apply complicated and custom-made scripts for data analysis.^54−56^ Analysis is also complicated in experiments where polydisperse droplets are generated via, for example, simple and quick vortexing.^19,39,50^ Yet, when assessing the current state and future direction of optical biodetection with droplet emulsion methods, as well as ways to improve accessibility to the general scientific public, polydisperse generation could be one part of the solution. In particular, this is true because analysis processing via deep learning algorithms is quickly advancing^19,72^ and the development of user-friendly and freely accessible pipelines from droplet image analysis is robust.^73−75^ Making droplet emulsion generation quick and simple, moreover, especially correlates with the current trend seen in the field of nucleic acid detection. The focus is shifting toward not only high-resolution single-cell analysis^11,76,77^ but also rapid and easy to use diagnostic and monitoring tools.^22,24,28,78−80^ Nevertheless, when addressing detection of extremely small molecules in complex matrices (e.g., neurotransmitters and electrolytes), options are restrictive due to current scientific technological limitations. Often such target detection currently still requires very specific labels and/or specific complex experimental setups with costly hardware.^37,42,43,46^ There is, however, no doubt that droplet emulsion methods provide prospective workflows and labeling options. The overall future perspective of optical biodetection is thus looking bright (no pun intended). This tutorial presented a diverse range of approaches that extend beyond any particular workflow, offering labeling options for others to plan or improve their research and hopefully opening new avenues that they can explore.

## Supporting Information Available

Overview of all optical biodetection studies and key workflow aspects discussed in this tutorial (ZIP)
