# Supplementary material for: A Guide to Biodetection in Droplets
Source: Anal Chem. 2024 Jun 6;96(24):9745–55. doi: 10.1021/acs.analchem.3c04282 (PMC11190884; doi:10.1021/acs.analchem.3c04282)
Supplement: Supplementary file 1 — ac3c04282_si_001.zip [file ac3c04282_si_001.zip › Cover Page.pdf]

Supporting Information (SI)

A guide to biodetection in droplets

Simona Bartkova[1]*, Marta Zapotoczna[2], Immanuel Sanka[1], Ott Scheler[1]

[1] Department of Chemistry and Biotechnology, Tallinn University of Technology (TalTech), Akadeemia tee 15, Tallinn 12618, Estonia
[2] Faculty of Biology, Biological and Chemical Research Centre, University of Warsaw, Żwirki i Wigury 101, 02-089 Warsaw, Poland
[§] Current address: Laboratory of Infection Biology, Biological and Chemical Research Centre, University of Warsaw, Żwirki i Wigury 101, 02-089 Warsaw, Poland

*Corresponding author email: simona.bartkova@taltech.ee

Table of contents

Description of Table S1……………………………………………………………………………………S3
Table S1 (XLS file)………………………………………………………………………….Separate XLS File

Description of Table S1

Table S1 provides an overview of all optical biodetection studies discussed in this tutorial, grouped by their study area (i) nucleic acid, (ii) cells, and (iii) biomolecules and their activity. Key experimental workflow aspects of each study are classified as following: (i) Aim, herein defined as the purpose of the study. (ii) Biosample, specified here as the biological sample wherein the target(s) of interest is located or sample which itself is the target of interest. (iii) Technique, meaning optical approaches that enable detection, measurement and evaluation of signal(s) from the target of interest. (iv) Target, biological target of interest in biosample that can be detected, measured, and evaluated, such as specific DNA/RNA sequence, phenotypic characteristic, biological and/or chemical reaction. (v) Label, which we term as substance(s) such as fluorophores, dyes, and antibodies, which are used to tag the target, and thereby facilitate target detection, measurement, and evaluation by producing an optical signal. (vi) Signal, which is a detectable and measurable optical signal produced by the label when the target is tagged, enabling evaluation of the aim of the study.
